# Sketch of the Life of Ambrose Parey

**Published:** 1830

**Authors:** J. M. Staughton


### WESTERN JOURNAL

OF THE

### MEDICAL AND PHYSICAL SCIENCES.

OCTOBER, NOVEMBER, AND DECEMBER, 1830.

### ESSAYS AND CASES.

ART.—I. *Sketch of the life and writings of* AMBROSE PAREY: By J. M. STAUGHTON, M. D.

AMBROSE PAREY was born at the town of Laval, in France, in 1509. Of his parentage and of his early history we know but little. After the elementary education requisite, he began the study of Surgery, and having passed through the usual course of reading and dissection, he entered as a dresser in the Hotel Dieu, at Paris, and resided during three years in that great school of practical knowledge. His ardent thirst after professional information, induced him while quite young to attend the armies of his country to the field of battle. It was not then customary, as now, for surgeons to be furnished to the armies by government, but each general officer brought his favourite surgeon with him, who was rewarded by that officer in particular, and by voluntary donations from the men. The recompense was precarious, and at best but small; consequently, men of inferior abilities only could be procured. It was nothing but Parey's enthusiastic love for his profession, that induced him to leave the good city of Paris, and expose himself to the difficulties, dangers, and privations, of a soldier's life. He made his first campaign in the army with which Henry II. invaded Savoy, and was in the special employ of the Lord Marshal Montjan, who commanded the infantry.

There was much hard fighting, and Parey saw many and desperate wounds. It was on this expedition that he accidentally discovered that there was no poisoning about gunshot wounds. It must be borne in mind that the use of gunpowder had but lately been introduced into warfare, and that surgeons were utterly unacquainted with the true nature of gunshot injuries.—The tendency to mortification which was found to prevail in them, was attributed to the effects of poison. Some surgeons affirming that the balls, and other missiles were actually poisoned. while others insisted that it was from the powder that these peculiar symptoms proceeded. The idea of poison once being established, no further search was made into the nature of these wounds, and a mode of treatment founded on this idea was practised.

I offer no excuse for the extract which I now make from his works, nor for the very numerous extracts which I shall make. My object is to present Parey as he was. Our principal facts respecting him are to be found only in his own works, and it is better that he should speak for himself.

"Now at that time I was a very raw soldier. I had never seen the dressing of a gunshot wound. It is true I had read in John de Vigo, that wounds made by weapons of fire did participate of venenosity by reason of the powder; and for their cure he commands to cauterize them with oil of Elder, scalding hot. Before I applied the said oil, knowing that such a thing would bring great pain to the patient, I was willing to know first how other surgeons did at the first dressing, which was to apply the said oil as scalding hot as possible into the wounds with tents and seatons, and I took courage to do as they did. At last I wanted oil, and was constrained, instead thereof, to apply a digestive of yolks of eggs, oil of roses, and turpentine. That night I could not sleep, fearing some accident from my not cauterizing, and that I should find those to whom I had not used the boiling oil, dead empoisoned, which made me rise very early in the morning to visit them, where beyond my expectations, I found those to whom I had applied my digestive medicine, to feel little pain, and their wounds without inflammation or tumour, having rested well. The others to whom was applied the boiling oil I found feverish, with great pain and tumour about the edges of their wounds. And then I resolved with myself never again so cruelly to burn poor fellows wounded by gunshot. See then how I learned to cure gunshot wounds—not from books."

He was with the army of Turin several years. We have a singular testimony of his abilities from an old physician, who, after the taking of the city, always called for Parey when any great surgical work was to be done, for he was delighted with the bold and spirited manner in which this young surgeon operated.

To the Segnieur Marechal Montjan, this old physician said on parting, "My Lord, you have a surgeon young in years, but old in experience and wisdom. Keep him carefully, for he will do you both service and honour." Parey himself tells this tale of his early days with great simplicity, but he adds, with much modesty and sterling sense, "But the good old man did not know that I had lived three years in the Hotel Dieu attending the sick and dissecting the dead."

From this time forward, Parey was engaged in the service of his country under various leaders, until his reputation reached the ears of the king of France, and in 1552 he was appointed surgeon in ordinary to Henry II. and subsequently he served the succeeding monarchs Francois II., Charles IX., and Henry III., as he himself informs us in the dedication of his works to the latter sovereign.

The distinguished reputation he gained as a military surgeon has never been equalled in former or succeeding ages. He was the soul of the army. To the implicit confidence with which they relied on his skill was added an unbounded personal love for his unvarying kindness and humanity and attention to the meanest soldier. When he went forth with them to battle, courage and determination shone in every face. The men felt that the angel of mercy was in their company. The shout was "What have we now to fear, our Ambrose is with us?"—on one occasion they refused to march till Parey was brought out to them.

At the siege of Mentz, where the chief nobility of the kingdom were shut up with the French army, while Charles V. with one hundred thousand troops lay around it, every difficulty and privation was experienced—surgeons were found in the city, but they were not capable of affording those services to the wounded that their desperate condition required. The sentiment was general that were Parey but with them all would be well. A solemn message was conveyed to the king of France, begging that Ambrose Parey might be sent to them. An Italian captain, for an immense reward, undertook to introduce him into the city, which was closely invested by the enemy; which service he performed with great danger. It was the dead of night when the illustrious surgeon entered. The governor was aroused from his slumbers, the bells were rung, and the trumpets sounded; and when on the succeeding morning he shewed himself on the ramparts, honors were paid to him such as no surgeon ever received. "Mentz was then the bulwark of France, and so perfect was the confidence of its brave defenders in Parey, that it has always been ascribed to the presence of this single man that they kept the city till the gallant army that lay around it perished beneath its walls."*

To exhibit the estimation in which he was held by the soldiery, and the incessant demand that was made on his time for surgical assistance, I will introduce a short quotation from his voyages and travels. "At the siege of Hedin, a council was held, where I was called to know if I would sign, as divers captains, gentlemen, and others had done, that the place should be rendered up. I made answer, that it was not possible to be held, and that I would sign; for the little hope that I had that we could resist the enemies, and also for the great desire I had to be out of this torment and hell; for I slept not either by night or day, by

*John Bell.

reason of the great number of the wounded. When I entered into one lodging, soldiers attended me at the door, to go and dress others at another lodging; when I walked out, there was striving who should have me; and they carried me like a holy body, not touching the ground with my feet, in spite of one another. Nor could I give the necessary attention to so great a number of wounded."

In reply to one of his adversaries who was trying to crush him, he gives a brief auto-biography, which must be considered simply an honest man's vindication of his fair fame: "Moreover, you say you will teach me my lesson in the operations of surgery. This, Sir, I think you cannot do, because I have not only learned them in my study, and by the hearing of many years. the lessons of Doctors of Physic, but as I said before in my epistle to the reader, I was resident the space of three years in the hospital of Paris, where I had the means to use and learn divers works of surgery upon various diseases, together with anatomy upon a great number of dead bodies, as oftentime I have made manifest in the medical school at Paris, and my good luck hath made me see much more; for being called to the service of the kings of France, (four of whom I have served,) I have been in company at battles, skirmishes, assaults, and the besieging of cities and fortresses; as also I have been shut up in cities with them that have been besieged, having it in charge to dress the wounded. Also, I have dwelt many years in this great and famous city of Paris, where, thanks be to God, I have lived in good repute among all men, and have not been esteemed the least among men of my profession, seeing there was not any cure, were it ever so difficult and great, where my hand and my counsel have not been required. Now seeing these things, how can you dare to say you will teach me to perform the operations of chirurgery, since you never went further than your study."

One important object I have before me is to present a rapid view of the improvements made by this great reformer of Surgery, in the various branches of his favourite science. He was decidedly a practical man, and he hesitated not to innovate on the rules of Surgery, whenever he thought innovation would eventuate in improvement. Much of the violent opposition he experienced is ascribed to the daring manner in which he ventured to modify the various operations.

In injuries of the head, he pointed out much more precisely than any former surgeon, the peculiar circumstances which call for the operation of the trephine, and so clearly did he understand the nature of these injuries, that there are very few surgeons of the present day who would hesitate to follow his indications. He is the first who pointed out the difficulty and danger in trepanning over the frontal sinuses, and demonstrated the mode of operating which we now practice. He made many improvements in the instruments employed in injuries of the head. His exfoliating trephine is a valuable instrument, and might, under certain circumstances, be advantageously employed at present. You will find in his works, an accurate plate and a correct description of that instrument called Hey's saw, for the revival of which, the late Mr. Hey, of Leeds, received so much deserved credit. He describes an instrument like a pair of compasses, one leg of which is constructed so as to cut while the other is placed in a hole in a metallic disc, by which a small segment of bone may be removed without the ablation of so large a portion as the trephine necessarily requires.

In cataract he couched, directing the lens to be kept under the needle while we can say a pater noster, to prevent its rising. He knew the milky cataract, but held the erroneous idea that it would grow hard by age; an idea, however, from which all modern surgeons are not exempt. He operated for Lagophthalmia and Entropeon much as we do. He invented a speculum, or ophthalmostat, as the modern German surgeons more properly call it, very much like those now found in our cases of eye instruments. His practice of opening the eye in hypopeon, though once discarded, is coming again into use. Artificial eyes of silver and gold, he describes to be painted in the encaustic style, and slipped under the lids.

In hydrocele, he practised the use of the seaton. He devised a new operation for incomplete fistula in ano. Salivary fistulæ he understood and cured. In paracentesis abdominis, he cautions against the too sudden abstraction of the fluid, as it sometimes proves fatal. In erysipelas, he knew the value of blisters, and has recorded their efficacy in many instances. In one case where the patient was so disfigured that the church excommunicated him as being touched by the finger of God with leprosy, he succeeded in restoring him to health, and of course to the perfect enjoyment of christain privileges. In dentistry, he draws all the parapharnalia of instruments that a modern dentist could need. We can form no very exalted opinion of the surgeons' skill at toothdrawing in his day, for he says, "I would have a toothdrawer expert, and diligent in the use of his instruments, for unless he knows cunningly and readily how to use them, he can scarce so carry himself but that he will force out three teeth at once, oftentimes leaving that untouched which caused the pain."

He is the first who practised cutting the gums of children in dentition, and performed it first on his own child with wonderful success. He describes artificial teeth, and the various modes of fixing them. In ranula, he knew the superiority of the actual cautery over the knife. He invented the obstetrical forceps, which Levret afterwards brought into use, and Levret does not hesitate to ascribe the honor of the invention to Parey.

I would ask with great confidence, is not the principle on which Hunter's operation for aneurism is founded, and which has confessedly been the means of saving hundreds of lives, contained in the following words from Parey: "above all, I advise the young surgeon to avoid opening aneurisms, if they are not very small. Cut the skin above; separate the artery from the surrounding parts; pass a needle with a strong thread at both ends of the wound. Then should the said artery be tied and cut, and the wound treated as a simple wound, letting the thread fall away of itself." In what more accurate words could John Hunter himself have described that operation for the invention of which he has received such unbounded applause?

There are two improvements which Parey made in the practise of Surgery, either of which would have ensured any surgeon a passport to an immortality of fame. I allude to the improvement already mentioned in the treatment of gunshot wounds, and to the substitution of the ligature for the cautery; as a mode of arresting hœmorrhage. He was in very truth the author of the mild practice in gunshot wounds. The success of his practice was not enough to drive the notion of poison out of the heads of his opponents, and he had need of courage and perseverance to fight against the adversaries which this new doctrine raised up against him. Joubert and Chaumet, united in following the steps of this illustrious reformer. But Gourmelin, Dalechamp, and Riolan, opposed the doctrine, and persecuted the man with a bitterness and malice scarcely to be conceived. The truth however prevailed, and Guillemeau contributed much to the triumph of the principles of his master, by adopting and propagating them in his writings.

With respect to the ligature as a mode of arresting hœmorrhage, I must be more particular ; for I must confess it was in investigating the history of the ligature that I became so much enamoured with the character of the unparallelled surgeon in question, that I resolved on adopting it as the subject of an essay.

I have no hesitation in ascribing to Ambrose Parey the honor of introducing the use of the ligature into surgical practice. I know that in taking this ground I may be met with doubts. I have examined the subject diligently and am prepared to give honor to whom honor is due. I am well aware, indeed I shall presently shew, that the idea of tying a string around a bleeding vessel was known to the predecessors of Parey. Parey, I repeat it, introduced the use of the ligature into surgical practice. That man is entitled to the honor of an invention who renders it useful to mankind. Who dares deny to our illustrious Fulton the honor of introducing steam navigation, and yet who does not know the numerous failures that his predecessors experienced in their attempts to make the thing feasible.

Among the old writers we find traces of the ligature, but we want the proof that it was ever practised to any extent ; for the notices we find are too slight to induce us to believe that it was ever a common practice. Moreover I fully believe that when Parey first used the ligature, he did not know that any one had ever mentioned it. Hear his own words : "I confess freely and with great sorrow that I practised in a different manner from what I now write, after the amputation of an arm or a leg. But why ? I had always seen those who were called on to perform such operations, immediately after the member was cut off, make use of several cauteries as well actual as potential to stop the flow of blood ; a thing very cruel and horrible to relate, and causing four out of six patients to die. I caution young surgeons to let alone such cruelty and inhumanity, the rather to follow my method of practice, which God has been pleased to put in my mind, without my ever having seen it practised, or heard or read of it." (Lib. xii. chap. 35.)

From this it is evident that Parey believed himself the inventor of the ligature. Indeed it would seem so, for the first two trials of it that he made were performed in the presence of his friend Stephen Lariviere, surgeon to the king, and other professional brethren equally worthy of confidence, taking care to keep the cauterizing irons in the fire, ready in case the ligature should prove insufficient. He did not publish the discovery till he was perfectly sure of the results, and then he never used any other mode of practice. It was soon adopted by the most distinguished practitioners in Paris. The Medical College, however, set itself in direct opposition to Parey's doctrine, and condemned his practice as cruel and dangerous. Gourmelin in particular denounced Parey and his new mode of cure with incredible malevolence and envy. I forbear to quote any of the expressions which he and his satellites used against this excellent man. The spirit in which this attack was made is the same with which the crafty and the selfish, at the present day, attempt to crush and bury every thing new and useful.

Parey, secure in the possession of a good conscience, and of universal respect, was in general regardless of the injurious epithets that were applied to him. Sometimes, however, he was touched with the taunts of his enemies, but he usually replies to them in a tone of pleasant irony. "You see now my young master," (the epithet he usually bestows on the learned Professor Gourmelin,) "my answers to your calumnies, and I

pray, if you are an honest man, to review and correct your book, and not to hold young chirurgeons in this error, by reading that you teach them to use hot irons, after the amputation of a limb to stay the flow of blood ; seeing there is another means not so cruel, and much more sure and easy. Moreover, if to-day, after an assault of a city where divers soldiers have had arms and legs shot off by cannon bullets, cutlass and other instruments of war, to stay the flow of blood if you should use hot irons, it would be needful to have a forge and much coals to heat them. The soldiers also would hold you in such horror for this cruelty, that they would kill you ; as in time past they did one of the chiefest chirurgeons in Rome."

Mention is made of the ligature in Celsus. Galen also enumerates it among the means of arresting hœmorrhage. but by both these authors it is thought inferior to the cautery ; of course the ligature is to be found casually mentioned in the works of the long train of servile copyists that followed. But that it was in common use does not appear from any of their writings.

During the course of the debate, Parey found that he was not the first to whom the idea had occurred, and that he had only made practicable what had previously been imagined. Renouncing without any feelings of mortification the character of the inventor, he contented himself with propagating the practice and defending the doctrine. It was one of the chief arguments which his adversaries employed against his practice, that it was modern, unknown to the Greeks and the Romans. He himself announced the fact of its being an old mode, and replies to Gourmelin, who had called him "carnifex" and compared him, because of his dissections, to the public executioner in the following terms : We must have it in mind that he was addressing a jealous, turbulent, and conceited professor. "Come sir, know a little more about your learned ancestors and render greater justice to your cotemporaries. You act the man of learning, and yet don't know that the ligature of the vessels was known to the ancients. You pretend to teach, and yet don't know that it was formerly practised in Italy. You make books, and they do nothing but retard the march of science, by perpetuating the most dangerous errors, and by discouraging those who aim at its advancement ; which of us most resembles the hangman, to whom you have been low enough to compare me, you who talk of nothing but boiling oil and red hot irons, or I who simply tie the vessels with a thread."

The event has proved ! The name of Gourmelin has sunk into oblivion with infamy and disgrace, while the character of Parey shines with increasing splendour.

Gourmelin indeed commenced his attack on Parey in virtue of his official station, as Dean of the Medical Faculty. By their direction he endeavored to suppress the writings of Parey, and in the end prosecuted him before the Court of Parliament. This attempt however failed ; for though the right to give an "imprimatur" to all medical works was vested in the College, yet when Parey appeared and pleaded the cause of his writings before the assembled wisdom of his judges, they considered him above the law, and gave him permission to publish his works, in spite of Dr. Gourmelin and the other learned professors. But Gourmelin found the work so well suited to his taste, that after his official duties had been performed he carried on the war on his own account, and of his own free will persecuted him with the utmost malevolence. In this service he was aided by his pupil Comperat, who was if possible, more wicked, stupid, and ignorant, than his master.

To appreciate the true value of the ligature as a mode of arresting the flow of blood, we should fully understand the method antecedently employed—after an amputation, it was the custom o apply a piece of iron heated to redness to the bleeding orifices of the arteries. By this process an eschar was formed, and the flow of blood arrested. In a vast majority of cases the application was successful for the time. But any one conversant with the functions of the living body, must know that the part was deprived of its vitality, and that it would come away on the 8th or 10th day, and consequently the mouth of the artery would be left open and a fresh bleeding would come on. In some cases however the artery became obliterated by the stagnation of the blood, and consequently a perfect cure followed. Yet by this mode, Parey tells us that four out of six died from bleeding after an amputation. This however is the most favorable manner in which the actual cautery is employed. But if any one were to see the drawings, in some old surgical book, of the fiery furnace, and the surgeon brandishing a huge red-hot iron and applying it to the whole face of the stump, while clouds of smoke and vapour arise, and were to see the distorted countenance with which the poor patient is represented as shrieking under the intolerable anguish of these horrid tortures, then could be formed some idea of the superior advantage of the ligature.

The large folio which contains the writings of Parey, is a curious compound. In the words of John Bell, "The friends of Parey assisted him in the book, and have mixed up with the precious grain of his surgery, straw, chaff, and stubble. It was suited to the grotesque taste of the age, and was then considered as a very learned work. Had Parey published his cases and surgical operations, the fruits of his experience in one small volume, his fame, like that of Fabricius Hildanus, would have gone down unblemished to posterity. The physicians of Paris, whom he had very unwisely employed to adorn his book, performed it in such a style, that it looked more like the work of the learned college and Dr. Gourmelin and his stupid pupil Comperat, than of the manly and sensible Parey. His observations are scattered through this monstrous folio, and yet that salt so sparingly sprinkled, has preserved the corruptible mass for more than two hundred years. These learned compilers have made his book what it is, a system of anatomy, a compendium of surgery, a dyspensary of drugs, chemical and galenical; a collection of cases, a history of travels and voyages, by sea and by land. By them the text of Parey has been adorned with the most incredible tales, and the margins of his book with the most uncouth drawings of birds and beasts, and comets and monsters, &c."

The book was written in French, but immediately after translated into Latin, and a few years saw it in every language of

Europe. The English edition is the work of Thomas Johnson, a surgeon of some eminence. It appeared in 1634. It was translated from the Latin, so that it has suffered a double translation, and a great loss it is too; for there is a fire and an energy of expression in the antiquated French of Parey, which the Latin copies do not possess, and which consequently is not to be found in the English. I regret that in most of the preceding extracts, I have been under the necessity of following the English copy, having only partial quotations from the French within my reach.

As a man, Parey bore a character the most exemplary. He died at the advanced age of 81, universally regretted. He was at once the chief surgeon and the familiar friend of four successive monarchs of France. Beloved and respected among the citizens, in high favor at court, idolized by the army, we find him still the same honest narrator of his own failings and errors, as when he first made the campaign of Turin, under his old friend Lord Marshall.

With true patriotism he refused to engage in the service of the enemies of his country. The fortune of war threw him on one occasion into the hands of the Spaniards as a prisoner. His first thought was to disguise himself, hoping to escape the enormous ransom that would be required were it known that the chief surgeon of the French king was prisoner. Impelled however by motives of humanity, he tendered his services to a Spanish officer who had been shot through the lungs, and though the case terminated fatally, he shewed such consummate skill, that an offer was made to receive him into the Spanish service. "The chief surgeon" says he, "took me apart, and told me if I would remain with him he would use me very well. I thanked him very kindly for the honor he did me; and told him that I had no desire to do any services to the enemies of my country. Then he told me I was a fool, and if he were a close prisoner as I, he would serve the *devil* to get his liberty. I told him flatly that I would not at all dwell with him." He soon after received his liberty as the reward of curing a distinguished Spanish officer of a varicose ulcer.

Parey was a protestant, a man of high and elevated piety. His usual expression when narrating the particulars of a case, is striking: "I dressed him and God cured him." His whole life exhibited the practical effects of Christian principles. Parey was a protestant, and yet he was the bosom friend of four sovereigns distinguished for their attachment to the established religion of France. Parey was a protestant, and yet he escaped the horrors of St. Bartholomew's day. That odious compound of selfishness, ignorance, and bigotry, Charles IX. preserved him alive, because of the interest he felt in securing to himself the command of his invaluable surgical attainments.

The Duke of Sully in his memoirs does not hesitate to ascribe to Parey's influence the order which was issued to arrest the hand of murder and assassination, after it had devastated Paris for two days. In a private interview Charles expressed himself to Parey as follows: I quote from Sully's memoirs—"The hour is now come," said the king, "when all France shall be of one religion." "Now, by the holy light of heaven, Sire," replied Parey, nothing alarmed, "I think you will never forget your promise to me, that there are four things you would not force me to do:—to enter again into my mother's womb—to go out in the day of battle—to leave your service—nor to go to mass." The king then took him aside and disclosed to him the troubles with which his soul was disquieted. "Ambrose," says he, "I know not how it is with me, but it goes so heavily, that within these three days I am as in a fever; indeed I am ill in mind and in body; sleeping and waking, the murdered Hugonots are ever before my eyes, with hideous faces, weltering in their blood! would to God the aged and the children had been spared!" The order for stopping the massacre, which was proclaimed on the following morning, was the result of this conversation.

I am sensible that in this attempt to point out the chief merits in the character of Parey, I have not done justice to the subject. A pen more ready, and a tongue more eloquent than mine, would find it inexhaustible.

Had I powers equal to the subject, I would hold him up as the individual in whom the qualities of a surgeon shine pre-emiently bright. I would present him as an operator, audax in securis, timidus in periculis. As an original genius, to whom persecution and opposition were only incentives to perseverance. I would describe him as the zealous and unwearied follower of nature, as the close and accurate observer of the protean forms of disease, as the bold and undaunted asserter of his own opinions, as the venerable patriarch of modern surgery.

What was surgery before the days of Parey! A servile following of the rules of Hippocrates, of Celsus, and of Galen.—As surgery must be founded on the immutable basis of anatomy, that cannot be good surgery which proceeds from men wholly ignorant of it. Hippocrates never dissected a human subject. Celsus is known to have compiled his work De Re Medica, from the writings of others, and he was not even a practitioner. And Galen, too, who lived in late days, undertook a long and perilous voyage to Alexandria, in Egypt, for the sole purpose of examining a skeleton. Here then was a man who preferred the knowledge he derived in the dissecting room and on the field of battle, to the antiquated commentaries of antiquated doctrines. He rose above the level of a surgeon's habitual pursuit. He gained the vantage ground of a higher knowledge. Lord Bacon, in his Novum Organon, speaking of such transcendant genius, well describes the elevated situation on which this great reformer stood, " Prospectationes fiunt a turribus, aut locis prealtis; et impossible est ut quis exploret remotiores interiores que scientiæ alicujus partes, si stet super plano ejusdem scientiæ, neque altiores scientiæ veluti speculam conscendat."

Parey speaks of what he learned from experience. " This I learned not from books," often occurs in his writings. And much did he learn from experience, for he could think with more profit than most men can read. The elementary education of this distinguished man was thorough, and in the usual routine of professional studies he was well grounded, and so was capable of profiting by his experience: for it is by the combination of knowledge and experience, that the medical character is formed. No man ever practised medicine or surgery with advantage to his patients and with reputation to himself, in virtue of his experience alone. Without sound, scientific knowledge, a medical man is more dangerous than an idiot brandishing a torch. It must not, however, be supposed, that Parey did not read. At his death, his library was found to bear the marks of incessant use, and the copy of Guy de Chauliac, at that period the best treatise on surgery, was found to have been so much used, handled, and worn, that it was in perfect tatters.

